# Either *fadD1* or *fadD2*, Which Encode acyl-CoA Synthetase, Is Essential for the Survival of *Haemophilus parasuis* SC096

**DOI:** 10.3389/fcimb.2017.00072

**Published:** 2017-03-15

**Authors:** Saixiang Feng, Chenggang Xu, Kaijie Yang, Haihong Wang, Huiying Fan, Ming Liao

**Affiliations:** ^1^Key Laboratory of Veterinary Vaccine Innovation of the Ministry of Agriculture, College of Veterinary Medicine, South China Agricultural UniversityGuangzhou, China; ^2^Key Laboratory of Protein Function and Regulation in Agricultural Organisms of Guangdong province, College of Life Science, South China Agricultural UniversityGuangzhou, China

**Keywords:** *Haemophilus parasuis*, acyl-CoA synthetase, FadD, fatty acid, quinolones

## Abstract

In *Haemophilus parasuis*, the genes *HAPS_0217* and *HAPS_1695* are predicted to encode long-chain fatty acid-CoA ligases (FACSs). These proteins contain ATP/AMP signature motifs and FACS conserved motifs that are homologous to those in *Escherichia coli* FadD (EcFadD). In this study, we demonstrate that *HAPS_0217* and *HAPS_1695* can functionally replace EcFadD in the *E. coli fadD* mutant JW1794, and were thus designated *fadD1* and *fadD2*, respectively. An evaluation of kinetic parameters indicated that FadD1 and FadD2 have a substrate preference for long-chain fatty acids. Moreover, FadD2 exhibited substrate inhibition in the presence of high concentrations of oleic acid. Single mutants of each of the *fadD* genes were easily constructed, whereas double mutants were not. These results were further confirmed using genomic site-directed mutagenesis, which supported the idea that *H. parasuis* requires either *fadD1* or *fadD2* for survival. The *fadD1* mutant exhibited slower growth than the wild-type strain SC096, and its complementation resulted in a restored phenotype. The wild-type strain did not grow on chemically defined medium without the addition of oleic acid, indicating that lipids are a vital nutrient for this bacterium. Additionally, strains with a disrupted *fadD1* gene also exhibited increased sensitivity to quinolone antibiotics, including levofloxacin, enrofloxacin, ciprofloxacin and nalidixic acid.

## Introduction

*Haemophilus parasuis* is a pathogenic bacterium of the upper respiratory tract in conventional pigs and the etiological agent of Glässer's disease, which is characterized by fibrinous polyserositis, arthritis, and meningitis. *H. parasuis* has emerged as a major cause of high mortality in swine that contributes to large economic losses in the pig industry worldwide (Oliveira and Pijoan, 2004). Intensive studies have attempted to identify the virulence-associated genes in *H. parasuis* (Costa-Hurtado and Aragon, 2013). However, several of the specific mechanisms underlying its pathogenicity remain to be investigated. Previous differential expression studies showed that exogenous fatty acid utilization enzyme FadD was a potential virulence factor in *H. parasuis* (Hill et al., 2003; Metcalf and MacInnes, 2007). Fatty acids are important metabolic intermediates as well as major components of phospholipids, which are essential for membrane formation in pathogenic bacteria (Zhang and Rock, 2008a). Because fatty acid biosynthesis is vital and energetically expensive, most pathogens use and incorporate extracellular fatty acids into their phospholipid membrane (Yao and Rock, 2015).

Over the past five decades, the fatty acid synthesis (FAS) and exogenous fatty acid incorporation pathways have been fully characterized in *Escherichia coli* (Rock and Jackowski, 2002). A highly conserved set of genes encode the enzymes that perform each of the individual steps in the FAS pathway (White et al., 2005). Alternatively, cells initiate the use of extracellular fatty acids by forming fatty acyl coenzyme A (acyl-CoA) using long-chain acyl-CoA ligase (FACS) (Black and DiRusso, 2003). Subsequently, phospholipids can be directly synthesized by acyltransferases using acyl-CoA (Zhang and Rock, 2008b). Another pathway uses acyl-CoA as a carbon source via β-oxidation (Weeks et al., 1969). Previous studies have shown that exogenous fatty acids are important participants in bacterial invasion and infection (DiRusso et al., 1999). For example, host cell phospholipase is required for *Salmonella enterica* serovar Typhimurium to invade epithelial cells (Falkow et al., 1992). Fatty acids are released by phospholipase and then imported and activated by the bacterial exogenous fatty acid transport system, resulting in an increase in the utilization of long-chain acyl-CoA (Pace et al., 1993).

The product of the *fadD* gene is a long-chain fatty acyl-CoA ligase that converts exogenous long-chain fatty acids (LCFAs) into acyl-CoA in bacteria (Black et al., 1992). In *Vibrio cholerae*, disrupting *fadD* results in attenuated virulence. In the *fadD* mutant, the expression of the major virulence genes in the ToxR regulon is repressed, and the membrane localization of the master regulator TcpP is impaired (Ray et al., 2011). The *fadD* mutant of *S. enterica* serovar Typhi has reduced *hilA* expression and invasiveness in HEp-2 cells, where *hilA* is a transcriptional regulator that is responsible for directly regulating the expression of genes, such as those involved in the type III secretory apparatus, that are required for invasiveness in *S. enterica* serovar Typhi (Lucas et al., 2000). Notably, *fadD* is not an essential gene for the survival of most bacteria, although it is important for pathogenic virulence activity in some bacteria. Pathogens can regulate FAS genes to supply endogenous fatty acids in an exogenous fatty acid-limited environment (DiRusso et al., 1999).

Here, we report the identification of two long-chain acyl-CoA ligases, FadD1 and FadD2, in *H. parasuis* SC096. These enzymes were confirmed to be responsible for the formation of long-chain fatty acyl-CoA. Either *fadD1* or *fadD2* is essential for the survival of *H. parasuis* SC096 grown in TSA medium containing bovine serum and NAD. The *fadD1* mutant exhibited an impaired growth phenotype in a chemically defined medium and was more susceptible to quinolone antibiotics.

## Materials and methods

### Bacterial strains, plasmids and growth conditions

The bacterial strains and plasmids used in this study are listed in Table 1. *E. coli* was grown in LB medium (10 g/l tryptone, 5 g/l yeast extract, and 10 g/l NaCl; pH 7.0) at 37°C following a routine protocol. *H. parasuis* was grown in Trypticase Soy Agar (TSA) (Oxoid, Hampshire, UK) supplemented with 0.002% nicotinamide adenine dinucleotide (NAD) (Sigma Aldrich, USA) and 5% inactivated bovine serum at 37°C in 5% CO_2_. The chemically defined medium was prepared according to a previously described protocol (Murphy and Brauer, 2011) with some modifications and had the following composition: 0.1 M NaCl, 2 mM K_2_HPO_4_, 2 mM KH_2_PO_4_, 4 mM Na_2_EDTA, 4 mM NH_4_Cl, 0.125 mM NaHCO_3_, 0.006 mM thiamine hydrochloride, 0.001 mM thiamine pyrophosphate, 0.008 mM calcium pantothenate, 0.02% nicotinamide adenine dinucleotide, 0.375 mM hypoxanthine, 0.45 mM uracil, 0.15 mM glutathione, 0.012 mM biotin, 5% glucose, 0.1 mM Fe(NO_3_), 2.5 mM MgCl_2_, 0.6 mM CaCl_2_, 6.25 mM Na acetate trihydrate, 1.125 mM L-alanine, 0.875 mM L-arginine hydrochloride, 0.2 mM L-asparagine, 3.75 mM L-aspartic acid, 0.35 mM L-cysteine hydrochloride hydrate, 0.15 mM L-cysteine, 7.5 mM L-glutamic acid, 0.35 mM L-glutamine, 0.225 mM glycine, 0.10 mM L-histidine, 0.225 mM L-isoleucine, 0.7 mM L-leucine, 0.35 mM L-lysine hydrochloride, 0.1 mM L-methionine, 0.15 mM L-phenylalanine, 0.45 mM L-proline, 0.475 mM L-serine, 0.425 mM L-threonine, 0.4 mM L-tryptophan, 0.4 mM L-tyrosine, and 0.525 mM L-valine, pH 7.2. When required, the medium was supplemented with kanamycin (30 μg/ml) and gentamicin (30 μg/ml) to grow *E. coli* and *H. parasuis* or erythromycin (10 μg/ml) to grow *H. parasuis* alone. Bacterial growth was determined by measuring the optical density at 600 nm.

**Table 1:** **Bacterial strains and plasmids used in this study**.

**Strain or plasmid**	**Relevant characteristic(s)**	**Source**
***E. coli*** **strains**		
DH5α	F^−^, ϕ80d/*lacZ*ΔM15, Δ(*lacZYA*-*argF*)169, *recA1, endA1, hsdR17*, Δ*phoA8, glnV44, deoR481, gyrA96*	Laboratory collection
BW25113	*lacI*^q^, *rrnB*_T14_, Δ*lacZ*_WJ16_, *hsdR514*, Δ*araBAD*_AH33_, Δ*rhaBAD*_LD78_	Datsenko and Wanner, 2000
JW1794	BW25113 *ΔfadD*::Kan^*r*^	Baba et al., 2008
BL21 (DE3)	F^−^, *ompT, hsdS*_B_ (${\text{r}}_{\text{B}}^{-}$ ${\text{m}}_{\text{B}}^{-}$), *gal, dcm*, (DE3)	Thermofisher
***H. parasuis*** **strains**		
*H. parasuis* SC096	Serovar 4 clinical isolate	Zhang et al., 2012
SF345 (Δ*fadD1*)	SC096 Δ*fadD1*::Gm^*r*^	This study
SF346 (Δ*fadD2*)	SC096 Δ*fadD2*::Kan^*r*^	This study
SF347 (Δ*fadD1*-c)	SC096 complemented Δ*fadD1* strain; Gm^*r*^, Kan^*r*^	This study
SF348 (Δ*fadD2*-c)	SC096 complemented Δ*fadD2* strain; Gm^*r*^, Kan^*r*^	This study
SF350	SC096 *fadD1*^T214A^*-*Em^*r*^	This study
SF351	SC096 *fadD1*^G216A^*-*Em^*r*^	This study
SF352	SC096 *fadD1*^T217A^*-*Em^*r*^	This study
SF353	SC096 *fadD1*^G219A^*-*Em^*r*^	This study
SF354	SC096 *fadD1*^E362A^*-*Em^*r*^	This study
SF355	SC096 *fadD1*^G216A^*-*Em^*r*^, Δ*fadD2*::Kan^*r*^	This study
SF356	SC096 *fadD1*^G219A^*-*Em^*r*^, Δ*fadD2*::Kan^*r*^	This study
**Plasmids**		
pMD19-T (simple)	T-vector, Amp^*r*^	Takara Inc
pK18mobsacB	Suicide and narrow-broad-host vector, Kan^*r*^	Schafer et al., 1994
pSF115	Kanamycin resistance cassette carrying complementation vector; Kan^*r*^	Zou et al., 2013
pET-28b	Kan^*r*^; expression vector	Novagen
pBAD24	Amp^*r*^; arabinose-inducible vector	Guzman et al., 1995
pBAD24m	Amp^*r*^; *Nco*I site of expression vector pBAD24 changed to an *Nde*I site	Zhu et al., 2013
p34S-Gm	Gentamicin resistance cassette carrying vector; Gm^*r*^	Dennis and Zylstra, 1998
pSF116	Gentamicin resistance cassette carrying complementation vector; Gm^*r*^	Zhou et al., 2016
pSF224	*fadD1* cloned into pMD19-T; Amp^*r*^	This study
pSF225	*fadD2* cloned into pMD19-T; Amp^*r*^	This study
pSF226	*fadD1* from pSF224 inserted between the *Nde*I and *Hin*dIII sites of pBAD24m; Amp^*r*^	This study
pSF227	*fadD2* from pSF224 inserted between the *Nde*I and *Hin*dIII sites of pBAD24m; Amp^*r*^	This study
pSF228	*fadD1* from pSF224 inserted between the *Nde*I and *Hin*dIII sites of pET28b; Kan^*r*^	This study
pSF229	*fadD2* from pSF224 inserted between the *Nde*I and *Hin*dIII sites of pET28b; Kan^*r*^	This study
pSF230	A disruption cassette containing gentamicin resistance cassette, the upstream and downstream sequences of *fadD1* in pMD19-T; Amp^*r*^, Gm^*r*^	This study
pSF231	A disruption cassette containing gentamicin resistance cassette, the upstream and downstream sequences of *fadD2* in pMD19-T; Amp^*r*^, Gm^*r*^	This study
pSF232	PCR-amplified *fadD1* cloned between the *Kpn*I and *Bam*HI sites of pSF116; Gm^*r*^	This study
pSF233	A fragment containing erythromycin resistance cassette and the intact *fadD1* gene with upstream and downstream sequences in pMD19-T; Amp^*r*^	This study
pSF234	carrying *fadD1* recombination cassette derived from pSF233 with a T214A mutation in FadD1; Em^*r*^	This study
pSF235	carrying *fadD1* recombination cassette derived from pSF233 with a G216A mutation in FadD1; Em^*r*^	This study
pSF236	carrying *fadD1* recombination cassette derived from pSF233 with a T217A mutation in FadD1; Em^*r*^	This study
pSF237	carrying *fadD1* recombination cassette derived from pSF233 with a G219A mutation in FadD1; Em^*r*^	This study
pSF238	carrying *fadD1* recombination cassette derived from pSF233 with a E362A mutation in FadD1; Em^*r*^	This study
pSF239	PCR-amplified *fadD2* cloned between the *Kpn*I and *Bam*HI sites of pSF115, Kan^*r*^	This study
pSF240	*fadD1*^T214A^ from pSF234 inserted between the *Nde*I and *Hin*dIII sites of pBAD24m; Amp^*r*^	This study
pSF241	*fadD1* ^G216A^ from pSF235 inserted between the *Nde*I and *Hin*dIII sites of pBAD24m; Amp^*r*^	This study
pSF242	*fadD1*^T217A^ from pSF236 inserted between the *Nde*I and *Hin*dIII sites of pBAD24m; Amp^*r*^	This study
pSF243	*fadD1*^G219A^ from pSF237 inserted between the *Nde*I and *Hin*dIII sites of pBAD24m; Amp^*r*^	This study
pSF244	*fadD1*^E362A^ from pSF238 inserted between the *Nde*I and *Hin*dIII sites of pBAD24m; Amp^*r*^	This study

### Complementation of an *E. coli* Δ*fadD* strain

A *fadD* mutant strain of *E. coli* (JW1794) was used for complementation studies (Baba et al., 2006). The *H. parasuis fadD* genes were amplified from the genomic DNA of the SC096 strain using the primers P1–P4, which contain *Nde*I and *Hin*dIII sites (as shown in Table 2). The PCR fragments were purified and cloned into the pMD19-T vector (Takara). The *fadD* sequences were confirmed by sequencing. The pSF226 (carrying *fadD1*) and pSF227 (carrying *fadD2*) plasmids were constructed by ligating *Nde*I-*Hin*dIII-digested fragments obtained from pMD19-T with the expression vector pBAD24m, which was digested with the same enzymes. The two recombinant vectors were introduced into the *E. coli* strain JW1794 for complementation. An empty vector was also transformed into JW1794 and used as a negative control in the complementation experiments. These strains were inoculated into M9 minimal medium supplemented with 0.1% fatty acid with Brij58 (0.02% arabinose was added if required), and the complementation results were determined after the cells were incubated for 72 h at 37°C.

**Table 2:** **Sequences of the PCR primers used in this study**.

**Primers**	**Primer sequences (5′–3′)**
P1 (*fadD1*-F for T)	CATATGGAAAAAATTTGGTTTGA
P2 (*fadD1*-R for T)	AAGCTTTTACAATTTTCCTTCCATCT
P3 (*fadD2*-F for T)	CATATGGCTTCTCTCGACTTCCA
P4 (*fadD2*-R for T)	AAGCTTATGAACGTTCATTAACTTAA
P5 (*fadD1*-Uf for mut)	ACCGCTTGTGAGATTGAGTTTAGTTCACA
P6 (*fadD1*-Ur for mut)	ATGTCAATTCGGGATCCGCGCCACATCTAAGGTACGTTCA
P7 (*fadD1*-Df for mut)	GATCGGCTTCGTCGACACGTTGTTGCGAGATGAGGAAGTT
P8 (*fadD1*-Dr for mut)	GTAACTCATAGATCGCATCA
P9 (Gm-F)	CGCGGATCCCGAATTGACATCGAATTGACATAAGCCTGTTC
P10 (Gm-R)	ACGTGTCGACGAAGCCGATCTCGGCTTGAAC
P11 (*fadD2*-Uf for mut)	ACCGCTTGTGATCGGCTTCGTCGACACGT
P12 (*fadD2*-Ur for mut)	ATGTCAATTCGGGATCCGCGGATCGGCTTCGTCGACACGT
P13 (*fadD2*-Df for mut)	GATCGGCTTCGTCGACACGTGATCGGCTTCGTCGACACGT
P14 (*fadD2*-Dr for mut)	GATCGGCTTCGTCGACACGT
P15 (Kan-F)	CGCGGATCCCGAATTGACATTTTTATGGACAGCAAGCGAA
P16 (Kan-R)	ACGTGTCGACGAAGCCGATCTCAGAAGAACTCGTCAAGAA
P17 (*fadD1*-F for comp)	CGCGTCGACAGTGTTGTGTACTACGGCAG
P18 (*fadD1*-R for comp)	CATGCATGCCTACAATTTTCCTTCCATCTGT
P19 (*fadD2*-F for comp)	CGCGTCGACCTTAAGAATACAGACAAACG
P20 (*fadD2*-R for comp)	CATGCATGCTTAAGTTAATGAACGTTCATTA
P21 (*fadD1*-R for PM)	CTGTGTTTTATATTTTTCTCGTTCATTTACAATTTTCCTTCCATCT
P22 (*fadD1*-Df for PM)	AGCTATAAATTATTTAATAAGTAATGAGAATAAGTGGTATATCT
P23 (Em-F)	ATGAACGAGAAAAATATAAAACACAG
P24 (Em-R)	TTACTTATTAAATAATTTATAGCT
P25 (T214A-F)	ATCTGGCGTTTCTTCAATATGCAGGCGGGACAACAGGAGTG
P26 (T214A-R)	ATATTGAAGAAACGCCAGAT
P27 (G216A-F)	GTTTCTTCAATATACAGGCGCAACAACAGGAGTGGCTAAAGG
P28 (G216A-R)	CGCCTGTATATTGAAGAAAC
P29 (T217A-F)	TTCTTCAATATACAGGCGGGGCAACAGGAGTGGCTAAAGGG
P30 (T217A-R)	CCCGCCTGTATATTGAAGAA
P31 (G219A-F)	ATATACAGGCGGGACAACAGCAGTGGCTAAAGGGGCAATGC
P32 (G219A-R)	CTGTTGTCCCGCCTGTATAT
P33 (E362A-F)	TGAAGGTTATGGTATGACCGCATGTTCCCCATTGATTGCGG
P34 (E362A-R)	CGGTCATACCATAACCTTCA
P35 (*fadD1*-R for RT)	AGTTGACCAGCGTACGCT
P36 (*fadD2*-R for RT)	TGACAGACCTGATCAAGT
P37 (*rplM*-R for RT)	TGCCACCTACATAGCCAG
P38 (*fadD1*-F for RT)	GCGATTGTCGTCGTGTCA
P39 (*fadD2*-F for RT)	TCGTTGGACTATTGCTGA
P40 (*rplM*-F for RT)	GTGACTGGTATGTAGTAG
P41 (*fadD1*-F for PCR test 1)	CCAGTGTTTACAGCCAGACA
P42 (*fadD1*-R for PCR test 1)	TAAGATCCGCTTGTGTGCGA
P43 (*fadD2*-F for PCR test 2)	GGTGATCGCTTAGACGATCA
P44 (*fadD2*-R for PCR test 2)	AATCATCTTGTTGTGCTAGG
P45 (*fadD1*-F for PCR test 3)	ATTGCCACACGCAGTGAGTT
P46 (*fadD1*-R for PCR test 3)	GGTCATACCATAACCTTCAA
P47 (*fadD2*-F for PCR test 3)	GGTGCGACAAATTGCTACTT
P48 (*fadD2*-R for PCR test 3)	TCTTCAGGCTTCTTGTAGTA
P49 (*fadD1*-F for PCR test 4)	ATGGAAAAAATTTGGTTTGA
P50 (*fadD2*-F for PCR test 4)	ATGGCTTCTCTCGACTTCCA
P51 (*ompP5*d-R for PCR test 4)	AGAATAATTGGTAACAAACCAATA

### FadD expression and purification

To produce the expression plasmids pSF228 (pET28b carrying *fadD1*) and pSF229 (pET28b carrying *fadD2*), cloning vectors (pMD19-T) carrying the above-constructed *fadD* genes were digested using *Nde*I and *Hin*dIII. The fragments were gel-purified and ligated into pET28b, which was digested using the same enzymes, to generate the expression vectors. The plasmids were then transformed into the *E. coli* BL21 strain. The respective FadD1 and FadD2 proteins were expressed at high levels and purified by growing the FadD expression strains at 37°C in LB medium. When the OD_600_ reached 0.8, the cultures were induced by the addition of 0.1 mM isopropyl-β-D-thio-D-galactoside (IPTG) and grown at 18°C for an additional 16 h prior to harvest. The cells were centrifuged and collected and then resuspended in lysis buffer containing 50 mM sodium phosphate, 300 mM NaCl, and 10 mM imidazole (pH 8.0). The cells were then lysed using ultrasonic disruption and centrifuged to remove unbroken cells and large debris. The supernatants were loaded onto a nickel-ion HisTrap HP affinity column in an ÄKTA Explorer FPLC system (GE). The column was washed with wash buffer (50 mM sodium phosphate, 300 mM NaCl, and 40 mM imidazole; pH 8.0), and the target proteins were eluted using elution buffer containing 300 mM imidazole. These protein solutions were dialyzed against lysis buffer without imidazole and analyzed using SDS-PAGE and MALDI-TOF.

### Measurement of fatty Acyl-CoA synthetase activity

Fatty acyl-CoA synthetase activity was monitored using Ellman's reagent as previously described (Kang et al., 2010) to detect the amount of free thiol. The reaction buffer mixture contained 150 mM Tris-HCl (pH 7.2), 10 mM MgCl_2_, 2 mM EDTA, 0.1% Triton X-100, 5 mM ATP, 0.5 mM reduced CoA, and fatty acid substrate (30–300 μM). The total reaction volume was 450 μl and included 10 μg of purified protein. Briefly, to perform the reaction, each mixture containing all of the components listed above (excluding CoA) was assembled, and 405 μl of the mixture was pre-incubated at 37°C for 3 min. The reaction was initiated with the addition of 45 μl of 5 mM reduced CoA (diluted to a final concentration of 0.5 mM), which was pre-incubated at 37°C for 3 min, quickly mixed, and incubated at 37°C throughout the reaction. Immediately after mixing, a recording was taken at the zero time point by removing 75 μl from the 450 μl reaction mixture and adding it to 600 μl of 0.4 mM 5,5′-dithiobis (2-nitrobenzoic acid) (DTNB, dissolved in 0.1 M potassium phosphate at pH 8.0), and the absorbance value at 412 nm was then measured. Subsequently, 75-μl aliquots of the reaction mixture were taken at 1-min intervals and mixed with DTNB to obtain additional measurements. The extinction coefficient of CoA was assumed to be 1.36 × 10^4^ M^−1^ cm^−1^. All reactions involving FadDs were repeated to obtain triplicate data for each fatty acid at each different concentration. The results were analyzed using Hanes-Woolf plots for various amounts of [S] against [S]/V for each concentration of fatty acid.

### Construction of *HpfadD* mutants and complementation strains

A suicide plasmid was constructed as follows to disrupt *H. parasuis fadD1*: DNA fragments located approximately 500 bp upstream and 500 bp downstream of *hpfadD1* (D1Up and D1Dn, respectively) were amplified from *H. parasuis* SC096 genomic DNA using the primers P5–P8 (Table 2). The marker gentamicin was amplified from p34S-Gm using the primers P9 and P10. All of the fragments were connected using overlap PCR with primers P5 and P8 and cloned into pMD19-T to obtain the plasmid pSF230, which carried the *fadD1* disruption cassette. The *fadD2* upstream and downstream fragments were amplified using the same method, except that the primers P11–P14 were used. The Kan^*r*^ gene was amplified from pK18mobsacB using the primers p15 and P16. These primers were ligated using overlap PCR with the primers P11 and P14 to obtain the plasmid pSF231, which carried a *fadD2* disruption cassette. These plasmids were transformed into SC096 to generate the *fadD1* or *fadD2* mutants SF345 and SF346, respectively, via natural transformation, as previously described (Zhang et al., 2012). The *fadD1* complementation strain was constructed as follows: an intact *fadD1* fragment with its native promoter was amplified with primers P17 and P18. The PCR product of the *fadD1* fragment was purified and digested with *Kpn*I and *Bam*HI. The fragment was then cloned into the complementation vector pSF115 (Zou et al., 2013) that was digested using the same enzymes to obtain the plasmid pSF232. The pSF232 plasmid was then transformed into SF345 (*fadD1* mutant) to generate the *fadD1* complementation strain SF347. A *fadD2* complementation plasmid was constructed using a similar method with the primers P19 and P20 and the complementation vector pSF116 (Zhou et al., 2016). The *fadD2* plasmid was transformed into SF346 (*fadD2* mutant) to obtain the *fadD2* complementation strain SF348.

### Site-directed mutagenesis and essentiality testing

To obtain a *fadD1* site-directed mutation strain, a mutation plasmid was constructed as follows: a fragment containing D1Up and intact *fadD1* was amplified using SC096 genomic DNA and the primers P5 and P21. Another fragment containing the intact *fadD1* gene and D1Dn was amplified using the primers P22 and P8. The erythromycin resistance gene Em^*r*^ was amplified using the primers P23 and P24. The fragments were connected using overlap PCR with the primers P5 and P8. The resulting PCR product was cloned into pMD19-T to obtain the template plasmid pSF233, which carried an intact *fadD1* gene containing upstream and downstream Em^*r*^ inserts at the end of *fadD1*. Five point mutations were generated using the primers P25–P34. For example, to place the T214A mutation in FadD1, an upstream fragment was amplified using the P5 primer and T214A point mutation primer P25 and using pSF233 as a template. The downstream fragment was amplified using the same template with primers P26 and P8. The two fragments were ligated using overlap PCR and cloned into pMD19T to obtain the plasmid pSF234, which carried the *fadD1* recombination cassette containing the T214A mutation in FadD1. The mutation vector was then transformed into SF345 (*fadD1* mutant) to generate the *fadD1*^T214A^ mutation strain SF350. The other four site-directed mutation strains (G216A, T217A, G219A, and E362A) were constructed using a similar method. To further confirm the essentiality of the *fadD* genes, *fadD2* disruption strains were constructed by natural transformation using *fadD1* site-directed mutation strains as recipients. The inability to obtain a mutation indicated that the selected mutation sites in FadD1 were essential for FadD1 activity and for supporting bacteria survival in a *fadD2* mutant.

### Analysis of fatty acid composition

A cellular lipid assay was performed as previously described (Zhu et al., 2013) with some modifications. Briefly, the *H. parasuis* strains were grown to mid-logarithmic phase in TSB medium containing bovine serum and NAD. The cells were harvested and washed three times with water. The cells were resuspended in 0.8 ml of water, and 1 ml chloroform and 2 ml methanol were then added. An additional 1 ml of water and 1 ml of chloroform were added after the solution was shaken for 1 h at room temperature. The suspensions were vortexed for 10 s and then centrifuged. The lower organic phase was extracted and washed twice with 2 M KCl and once with water and then dried in nitrogen. The phospholipids were dissolved in 1.2 ml of dry methanol, and 0.2 ml of 25% (vol/vol) sodium methoxide (Sigma) was added. A 1.2-ml volume of 2 M HCl was added after the solution was incubated for 30 min at room temperature. The fatty acid methyl esters were extracted using petroleum ether. The extraction agent was removed using a stream of nitrogen, and the samples were analyzed using gas chromatography (GC)-mass spectrometry in an Agilent 7890-5975C. The GC was equipped with a (5%-phenyl)-methyl polysiloxane HP-5MS column (60 × 250 × 0.25 μm). A chromatogram was produced by initially holding the oven temperature at 110°C and then increasing the temperature to 140°C at a rate of 5°C/min. The temperature was then held for 5 min, increased at a rate of 5°C/min to 180°C, and held for 5 min at 180°C. The temperature was increased to 250°C at a rate of 5°C/min and held for 5 min. Finally, the temperature was increased to 280°C at a rate of 5°C/min and held at 280°C for 5 min. The following conditions were used for mass detector: capillary direct interface temperature, 260°C; ionization energy, 70 eV; and mass range, 35–550 amu. Helium was used as the carrier gas (flow rate, 1.5 ml/min). The components in essential oils were identified using the NIST11 MS data library.

### RNA extraction and quantitative real-time PCR analyses

The *H. parasuis* strains were cultured to mid-logarithmic phase in TSB medium containing heat-inactivated newborn bovine serum and NAD. Total RNA was extracted using a Bacterial RNA Isolation Kit (Omega). The RNA was adjusted to a concentration of 200–500 ng/μl (measured using a NanoDrop 8000, Thermo Scientific), and all samples were reverse-transcribed using a PrimeScript™ RT Reagent Kit (Takara) with the specific primers P35, P36, and P37. Quantitative real-time PCR analyses were performed using an Applied Biosystems PRISM model 7500 Sequence Detection system with SYBR® Premix Ex Taq™ (Takara). The results were relatively quantified using the comparative cycle threshold method. The endogenous internal control *rplM* was used for sample normalization, as previously described (Zhou et al., 2016). The amplification program was as follows: 30 s at 95°C followed by 40 cycles at 95°C for 5 s and 60°C for 34 s. Three independent experiments were performed for each strain, and three technical replicate RT-PCRs were performed for each sample.

### Serum bactericidal assay

The serum bactericidal assays were performed using porcine serum as previously described (Zou et al., 2013). Porcine serum stored at −80°C from four healthy piglets (3–4 weeks old) from a farm free of Glässer's disease collected in previous work (Zhou et al., 2016) was used for this study. The piglets were free of Glässer's disease. The serum was filter-sterilized (0.22 μM), and aliquots were stored at −80°C. The controls were performed using serum samples that were heated at 56°C for 30 min. Wild-type and recombinant *H. parasuis* strains were grown to mid-logarithmic phase. A 100 or 400-μl volume of fresh porcine serum or heat-treated serum was mixed with 100 μl of a diluted bacterial suspension (10^8^ CFU) to achieve a final concentration of 50 or 80% serum. The mixture was then incubated at 37°C for 1 h. The bacteria were serially diluted and plated on TSA plates containing heat-inactivated newborn bovine serum and NAD. To quantify the results, the plates were incubated for 24 h at 37°C in an atmosphere containing 5% CO_2_. The percent survival was calculated as the ratio of the number of colonies grown from fresh serum to the number grown from heat-treated serum. Three independent experiments were performed for each *H. parasuis* strain.

### Antimicrobial susceptibility testing

To control the inoculum volume, the antimicrobial susceptibility of all of the *H. parasuis* strains was determined using the agar dilution method with an antibacterial determiner (SAKUMA, Japan). The results were interpreted according to the Clinical and Laboratory Standards Institute (CLSI) guidelines. The MIC value was defined as the lowest concentration that resulted in no visible colonies. The reference strains *Actinobacillus pleuropneumoniae* ATCC 27090 and *Escherichia coli* ATCC 25922 were used as quality controls for the MIC determination.

### Statistical analysis

Student's *t*-test was used to determine the significance of differences. A *p*-value < 0.05 was considered statistically significant.

## Results

### Two predicted Acyl-CoA synthetase genes in *H. parasuis*

**Figure 1:**
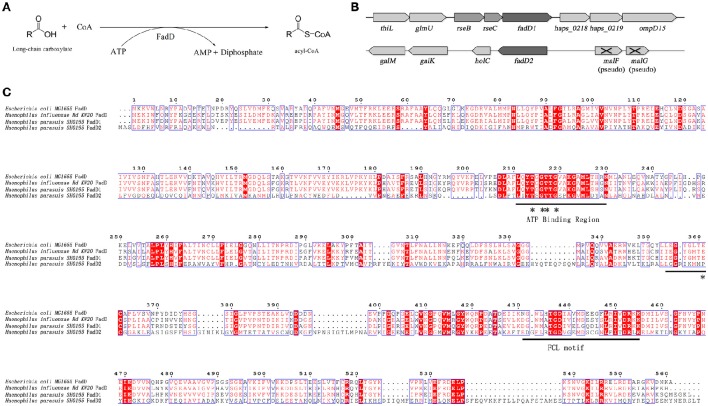
**The acyl-CoA synthetase (FACS) reaction, organization of the ***fadD*** genes clusters, and alignment of ***H. parasuis*** FadDs with homolog of ***E. coli*** and ***H. influenza***. (A)** The chemical equation of acyl-CoA synthetase reaction. **(B)** Organization of *H. parasuis fadDs* clusters. The filled arrows indicate the relative size and transcriptional direction of the genes. The numbers and names below the arrows indicate the gene annotations in the *H. parasuis* SH0165. The *resB* and *resC* genes encode cytochrome C biosynthesis-related proteins. The gene *holC* encodes DNA polymerase III chi subunit. **(C)** Alignment of *H. parasuis* FadDs with homologous of *E. coli* and *H. influenza*. The ATP/AMP and FACS motifs are indicated by underlines. Sites of experiment confirmed in *E. coli* are denoted by asterisks.

In this study, two *H. parasuis* genes, *fadD1* (*HAPS_0217*) and *fadD2* (*HAPS_1695*), were annotated and found to encode homolog of *E. coli* FadD, which is a FACS. FACSs belong to the ANL superfamily of adenylating enzymes. Members of this family catalyze two partial reactions: the first is the adenylation of a carboxylate to form an acyl-AMP intermediate, which is followed by a second reaction that usually leads to the formation of a thioester (Gulick, 2009). The reaction product of FadD is acyl-CoA (Figure 1A). A sequence comparison of members of the FACS protein family revealed the presence of an ATP-AMP signature motif and a FACSs conserved motif (Black and DiRusso, 2003).

*HpfadD1* is located in a chromosomal cluster with *resB* and *resC* (a putative cytochrome c biogenesis-associated protein), whereas *HpfadD2* is located at another locus and is likely to be monocistronic (Figure 1B). The gene cluster structures of both genes shared no similarity with the *E. coli fadD* locus. An alignment of the FadD homolog revealed that HpFadD1 was 65% identical to *E. coli* FadD and that the ATP/AMP and FACS motifs were conserved between them (Figure 1C). In contrast, HpFadD2 shared no similarity with EcFadD when analyzed using BLAST but was 22% identical to *E. coli* FadK, a short-chain acyl-CoA synthetase (Morgan-Kiss and Cronan, 2004). Interestingly, HpFadD2 was 25% identical to HpFadD1 and contained an ATP/AMP motif. This analysis indicates that FadD1 and FadD2 may have acyl-CoA synthetase activity.

### *H. parasuis fadD1* and *fadD2* functionally replace *E. coli fadD In vivo*

We used an *E. coli* complementation system to determine whether FadD1 or FadD2 functions as an acyl-CoA synthetase. The *E. coli* JW1794 strain is a *fadD* mutant that cannot survive in minimal medium when exogenous fatty acid is the sole source of carbon (Baba et al., 2006). This deficiency in *fadD* can be restored by a plasmid carrying the gene encoding acyl-CoA synthetase. Hence, *fadD1* and *fadD2* were cloned into the arabinose-inducible vector pBAD24m to produce the expression constructs pSF226 and pSF227, respectively. These plasmids were then transformed into the *E. coli* strain JW1794. The resulting transformants were tested on M9 medium plates supplemented with different fatty acids as the sole carbon source.

**Figure 2:**
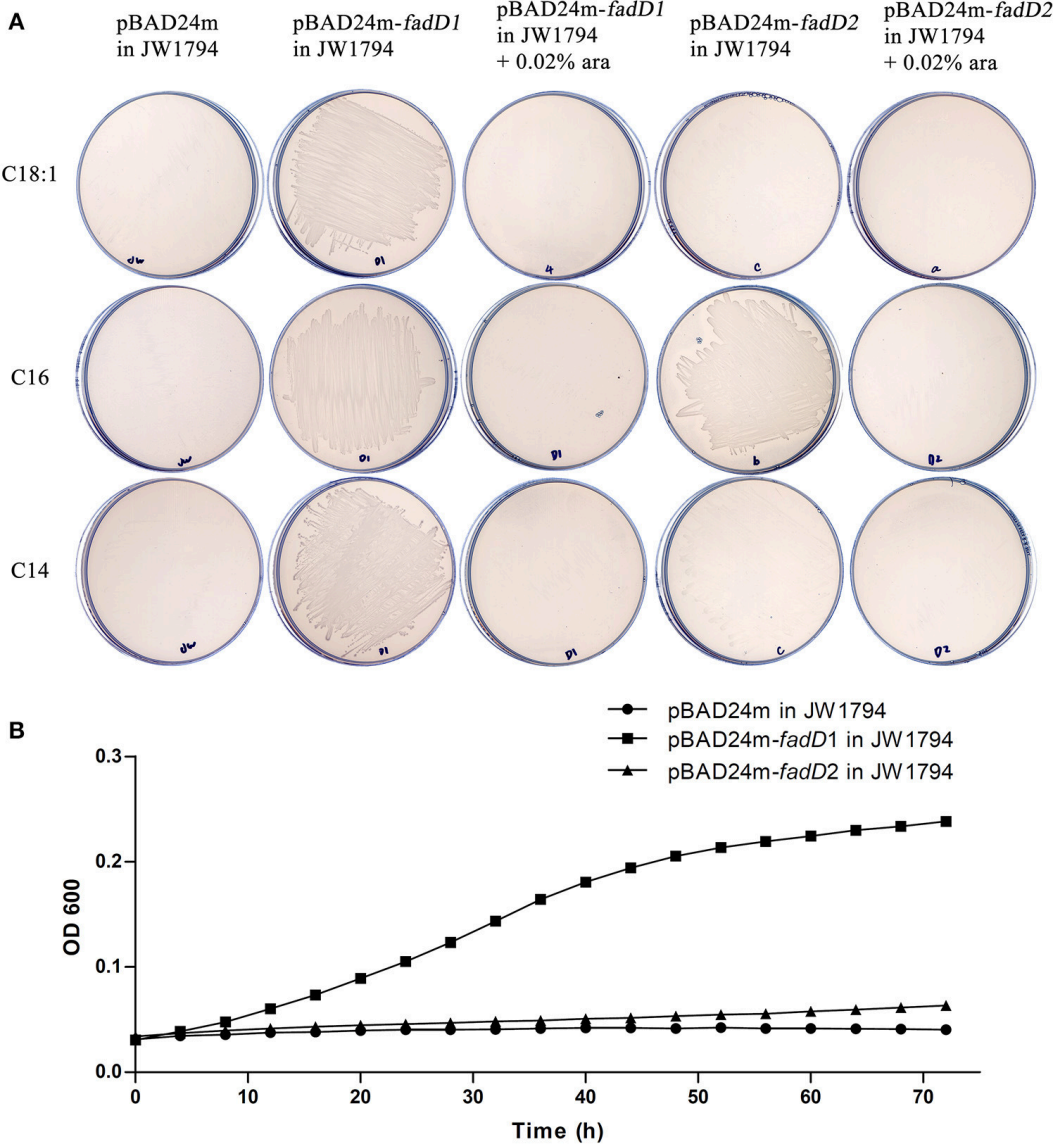
**Expression of ***H. parasuis*** FadDs restored growth of an ***E. coli fadD*** mutant JW1794. (A)** Transformants of *E. coli fadD* mutant JW1794 were grown on M9 minimal medium plates with various fatty acids as the sole carbon source. Growth was tested in either the presence or the absence of arabinose. The strains tested were JW1794 carrying plasmids pSF226 encoding *fadD1*, pSF227 encoding *fadD2*, respectively, or the vector plasmid, pBAD24m. Ara, arabinose; C14:0, myristic acid; C16:0, palmitic acid; C18:1, oleic acid. **(B)** Growth curve of JW1794 transformants grown in M9 broth with oleic acid as the sole carbon source. The strains tested were JW1794 carrying plasmids pSF226 encoding *fadD1*, pSF227 encoding *fadD2*, respectively, or the vector plasmid, pBAD24m. The data presented are averages of three independent experiments, and error bars represent standard deviations.

An examination of the growth of the complementation strains incubated on M9 minimal medium plates revealed that the JW1794 strain carrying *fadD1* grew on various fatty acids, whereas JW1794 carrying *fadD2* grew only on C16:0 (Figure 2A). These strains grew only in the absence of the inducer arabinose, indicating that high levels of expression of either FadD may be toxic, consistent with previous findings (Bi et al., 2014). To confirm that this phenotype was consistent when these strains were grown in M9 broth, we determined the growth curves of these strains. We found that the *HpfadD2* complementation strain survived when grown on minimal medium in which oleic acid was the sole carbon source, although it grew quite slowly compared with the *HpfadD1* complementation strain (Figure 2B, the doubling times of the *HpfadD1* and *HpfadD2* complementation strains were 13.38 ± 2.16 h and 37.36 ± 12.51 h, respectively, *P* < 0.05). Altogether, these results indicate that FadD1 and FadD2 have acyl-CoA synthetase activity *in vivo*.

### Purification and characterization of the FadD1 and FadD2 proteins

**Figure 3:**
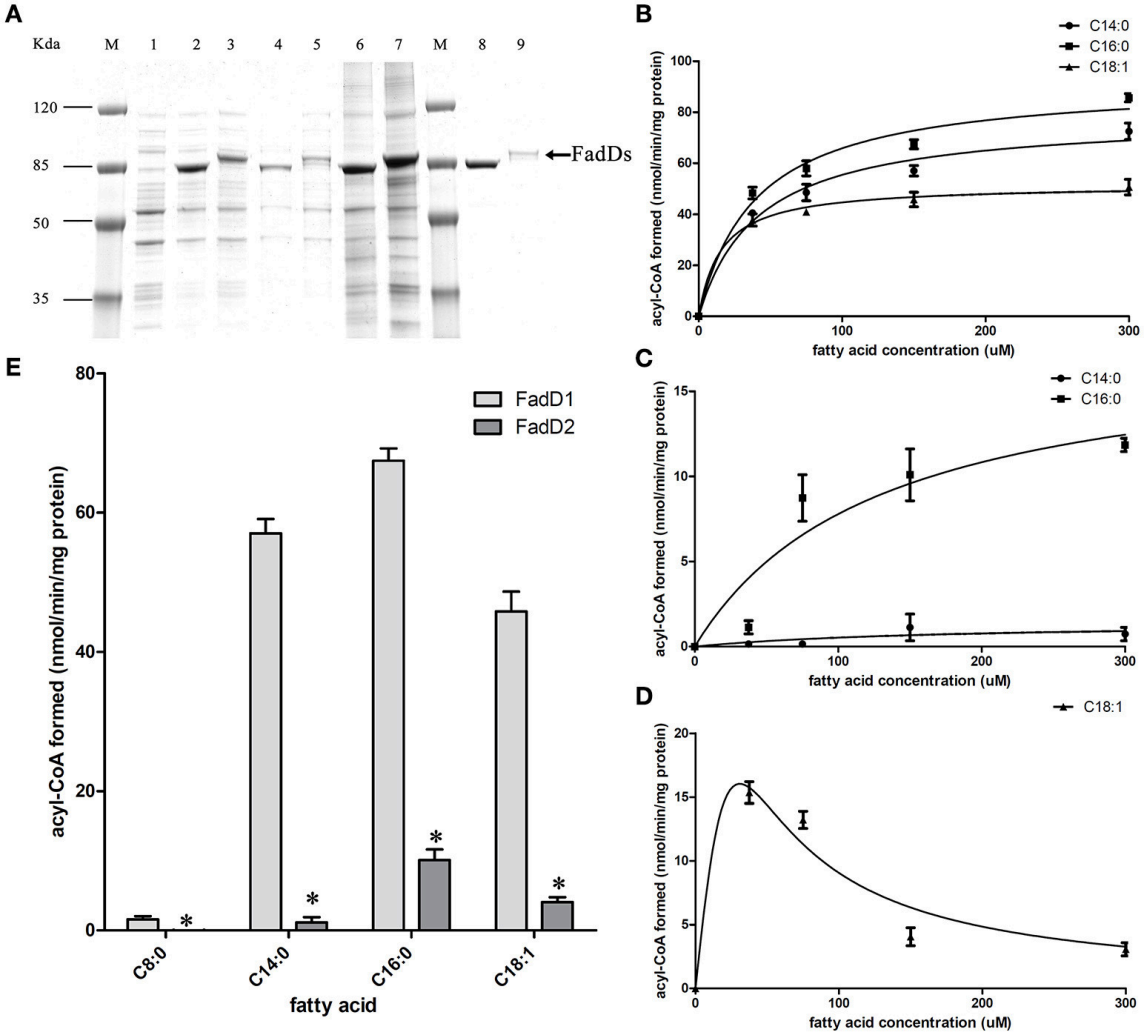
**Purification and biochemical characterization of the two ***H. parasuis*** FadDs. (A)** Purification of *H. parasuis* SC096 FadD1 and FadD2 by native nickel-chelate chromatography. Lane M, molecular mass markers; lane 1–3, proteins from whole cells extraction of BL21 with the vector plasmid pET28b, FadD1, and FadD2 expression strains; lane 4 and 5, supernatant of lysate from FadD1 or FadD2 expression strain; lane 6 and 7, centrifuged precipitation of lysate from FadD1 or FadD2 expression strain; lane 8, purified FadD1; lane 9, purified FadD2. **(B,C)** Kinetics of FadD1 and FadD2 activity was determined using fatty acids of different concentrations (30–300 μM) as the variable substrates. **(D)** Kinetics of FadD2 inhibition with oleic acid was determined using variable concentrations (30–300 μM) of oleic acid as the fatty acid substrates. **(E)** Activities comparison of FadD1 and FadD2 were performed using 150 μM oleic acid as a fatty acid substrate. The error bars of all the experiments represent the mean ± S.D. (*n* = 4). The asterisks indicated that activity of FadD1 was statistically different (*P* < 0.01) from that of the FadD2 as judged by the Student's *t-*test.

We produced His-tagged proteins to confirm the substrates of the two FACSs. FadD1 and FadD2 were expressed at high levels in *E. coli* grown in low-temperature induction conditions. The expression levels of the two enzymes were equivalent in whole cell protein extracts, but after ultrasonication and centrifugation, the lysed supernatant of the FadD2-expressing strain contained less of the target protein. Non-transparent inclusion bodies were not observed, indicating that FadD2 may be localized mainly in particulate fractions, as described in previous investigations that showed that EcFadD was located in both the soluble and particulate fractions of bacterial cells (Kameda and Nunn, 1981). The proteins were purified using nickel chelate chromatography in an AKTA explorer FPLC system. Target proteins were detected using SDS-PAGE (Figure 3A) and confirmed by mass analysis (Figure S1).

The fatty acid substrate specificity of each FadD was determined *in vitro*. FadD1 exhibited a high level of activity on C14:0, C16:0, and C18:1, with high *V*_max_ (78.42, 92.11, and 51.64 nanomoles of acyl-CoA formed/min/mg of protein, respectively) and low *K*_m_ values (41.48, 39.90, and 15.64 μM, respectively) when the substrate ranged from 30 to 300 μM (Figure 3B and Table 3). FadD2 showed clear activity only on C16:0 (17.78 nanomoles of acyl-CoA formed/min/mg of protein for *V*_max_, 128.30 μM for *K*_m_), and its level of activity was very low on C14:0 (1.45 nanomoles of acyl-CoA formed/min/mg of protein for *V*_max_, 173.40 μM for *K*_m_) (Figure 3C and Table 3). Moreover, oleic acid in different concentrations had distinct substrate inhibition effects on FadD2. A higher concentration of C18:1 resulted in lower FadD2 activity (Figure 3D). This may explain why the JW1794 strain carrying FadD2 could not grow on C18:1 (Figure 2A). The fact that FadD1 had higher *k*_cat/_*K*_m_ values on all selected FAs indicated that FadD1 had a higher level of activity than FadD2 (Table 3). These results are in accordance with the *E. coli* complementation testing results (Figure 2). FadD1 and FadD2 are long-chain FACS, as they both have higher LCFA (long-chain fatty acid) and MCFA (medium-chain fatty acid) activity than SCFA (short-chain fatty acid, such as C8:0) activity (Figure 3E). Therefore, FadD2 is not a short-chain FACS as FadK is in *E. coli*, although they share some homology.

**Table 3:** **Kinetic properties of FadD1 and FadD2 with various substrates**.

**FACS**	**Substrates**	**Kinetic parameters**^a^			
		***V*_max_^b^**	***Km***^c^	***k*_cat_^d^**	***k*_cat/_*Km^e^***
FadD1	C14:0	78.42	41.48	0.083	2.01
	C16:0	92.11	39.90	0.098	2.45
	C18:1	51.64	15.64	0.055	3.51
FadD2	C14:0	1.45	173.40	0.002	0.01
	C16:0	17.78	128.30	0.020	0.16
	C18:1	ND	ND	ND	ND

a *Kinetic constants (K_m_ and V_max_) defined using the Hanes-Woolf plot*.

b *Nanomole of acyl-CoA formed/min/mg of protein*.

c *μM of FA*.

d *s^−1^; determined using MW of FadD1(63684) and FadD2 (68500)*.

e *mM^−1^ s^−1^*.

### Either *fadD1* or *fadD2* is essential for *H. parasuis* SC096 survival

**Figure 4:**
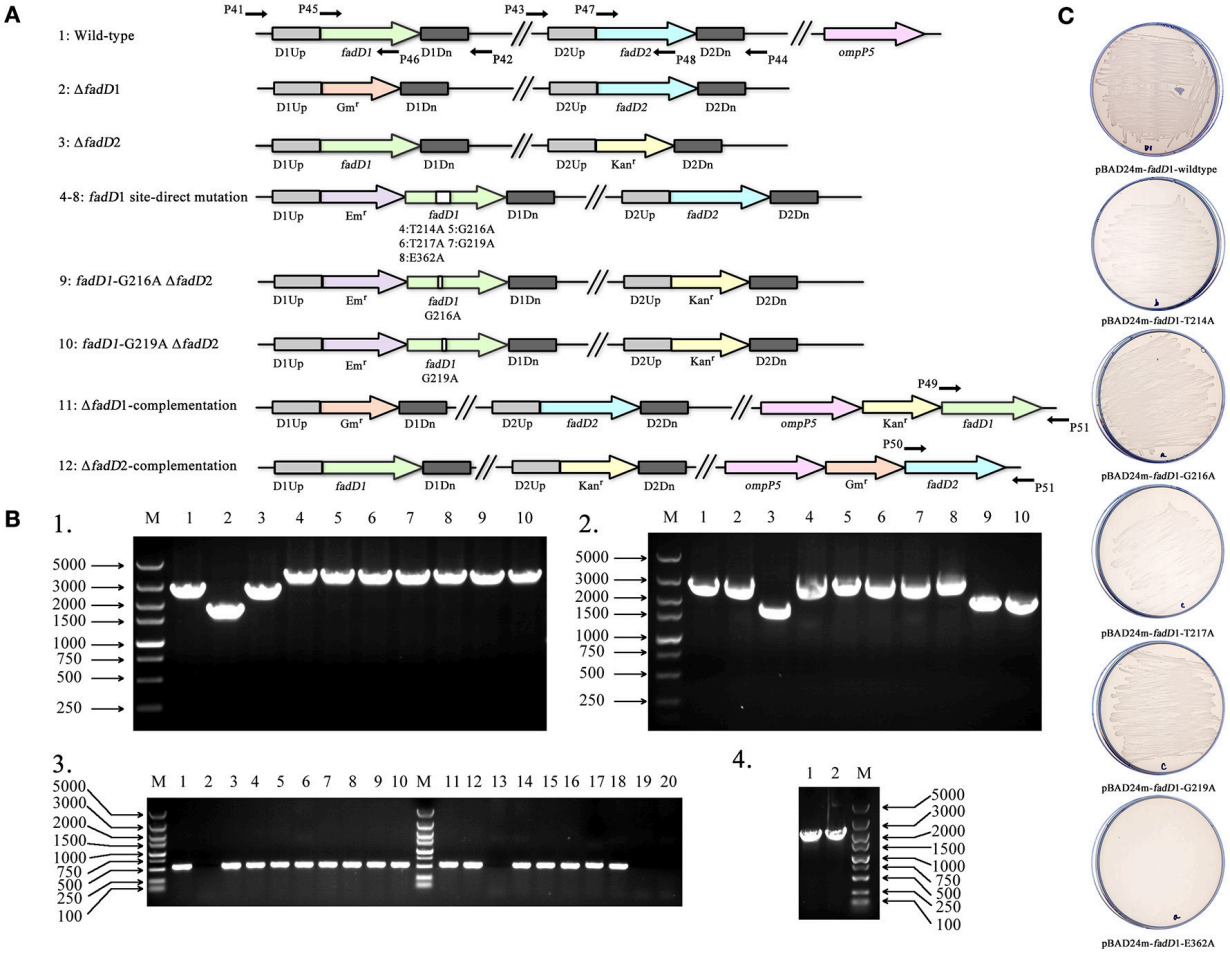
**Construction of in-frame deletion and site-direct mutants of ***fadD*** genes in ***H. parasuis***. (A)** Schematic diagram of mutants constructed in this study. Kan^*r*^, kanamycin-resistant gene; Gm^*r*^, gentamicin-resistant gene; D1Up, upstream sequence of *fadD1*; D1Dn, downstream sequence of *fadD1*; D2Up, upstream sequence of *fadD2*; D2Dn, downstream sequence of *fadD2*; T214A, threonine of the site 214 substituted to alanine in FadD1; G216A, glycine of the site 216 substituted to alanine in FadD1; T217A, threonine of the site 217 substituted to alanine in FadD1; G219A, glycine of the site 219 substituted to alanine in FadD1; E362A, glutamic acid of the site 362 substituted to alanine in FadD1. **(B)** Identification of mutations by PCR. Part 1 showed the PCR analysis of *fadD1* region in all 10 strains using primers P41 and P42 as shown in panel **A** (lane 1, SC096; lane 2, Δ*fadD1*; lane 3, Δ*fadD2*; lane 4, *fadD1*^T214A^; lane 5, *fadD1*^G216A^; lane 6, *fadD1*^T217A^; lane 7, *fadD1*^G219A^; lane 8, *fadD1*^E362A^; lane 9, *fadD1*^G216A^–Δ*fadD2*; lane 10, *fadD1*^G219A^–Δ*fadD2*). Part 2 showed the PCR analysis of *fadD2* region using primers P43 and P44 (lane 1, SC096; lane 2, Δ*fadD1*; lane 3, Δ*fadD2*; lane 4, *fadD1*^T214A^; lane 5, *fadD1*^G216A^; lane 6, *fadD1*^T217A^; lane 7, *fadD1*^G219A^; lane 8, *fadD1*^E362A^; lane 9, *fadD1*^G216A^–Δ*fadD2*; lane 10, *fadD1*^G219A^–Δ*fadD2*). The in-frame region of *fadD1* and *fadD2* were detected using primers P45–P48 as shown in Part 3 (lane 1, SC096; lane 2, Δ*fadD1*; lane 3, Δ*fadD2*; lane 4, *fadD1*^T214A^; lane 5, *fadD1*^G216A^; lane 6, *fadD1*^T217A^; lane 7, *fadD1*^G219A^; lane 8, *fadD1*^E362A^; lane 9, *fadD1*^G216A^–Δ*fadD2*; lane 10, *fadD1*^G219A^–Δ*fadD2*; lane 11, SC096; lane 12, Δ*fadD1*; lane 13, Δ*fadD2*; lane 14, *fadD1*^T214A^; lane 15, *fadD1*^G216A^; lane 16, *fadD1*^T217A^; lane 17, *fadD1*^G219A^; lane 18, *fadD1*^E362A^; lane 19, *fadD1*^G216A^–Δ*fadD2*; lane 20, *fadD1*^*G*219*A*^–Δ*fadD2*). Part 4 confirmed the complementation strain using primers P49–P51 as shown in panel **A** (lane 1, Δ*fadD1-*complementation and lane 2, Δ*fadD2-*complementation). **(C)** Transformants of *E. coli fadD* mutant JW1794 were grown on M9 minimal medium plates with oleic acid as the sole carbon source. The strains tested were JW1794 carrying plasmids pSF226, pSF240, pSF241, pSF242, pSF243, or pSF244 encoding *fadD1, fadD1*^T214A^, *fadD1*^G216A^, *fadD1*^T217A^, *fadD1*^G219A^, or *fadD1*^E362A^, respectively.

To determine the physiological functions of the two FACS in *H. parasuis, fadD1*, or *fadD2* mutants were constructed using natural transformation, as described in the methods section (Figure 4). However, double mutants were not produced when the *fadD1* disruption vector was transformed into the *fadD2* mutant or when the *fadD2* disruption vector was transformed into the *fadD1* mutant. These transformation experiments were performed more than five times each; however, no positive colonies were obtained. Notably, the complementation strains of Δ*fadD1* or Δ*fadD2* were obtained as described in the methods section (Figure 4 and Figure S2). These results indicated that the natural transformation ability of the Δ*fadD1* and Δ*fadD2* strains was not significantly attenuated.

In a previous study, residue substitutions were introduced in the ATP/AMP signature motif of *E. coli* FadD to identify the specific residues that are critical for its activity (Weimar et al., 2002). To further determine whether *fadD1* or *fadD2* is essential for *H. parasuis* survival, we performed genomic site-directed mutagenesis of five conserved amino acids in the ATP/AMP motif of FadD1. Schematics for the constructed strains are shown in Figure 4A. All of the strains were confirmed by PCR and sequencing (Figure 4B). First, the *fadD1*^T214A^, *fadD1*^G216A^, *fadD1*^T217A^, *fadD1*^G219A^, and *fadD1*^E362A^ strains were constructed. Subsequently, all site-directed mutants were naturally transformed with pSF231 (a *fadD2* disruption vector) to generate double-mutant strains. However, only *fadD1*^G216A^–Δ*fadD2* and *fadD1*^G219A^–Δ*fadD2* were obtained (Table 4). Moreover, the growth of *E. coli* JW1794 cells carrying plasmids encoding the mutant proteins (genes with point mutations contained in the expression vector pBAD24m) was also tested on oleate (Figure 4C). This assay demonstrated that FadD1^G216A^ or FadD1^G219A^ exhibited activity that was sufficient to support a similar level growth as that of JW1794 carrying wild-type FadD1 on minimal medium when oleic acid was the sole carbon source. Growth was summarized for of all of the complementation strains derived from the *E. coli* JW1794 *fadD* mutant in Table 5. In conclusion, these data indicated that a *fadD2* mutant can be generated only when the strain has sufficient FadD1 activity. Hence, either *fadD1* or *fadD2* is required for the survival of this bacterium.

**Table 4:** **Construction of ***fadD*** mutants in this study**.

**Mutation vector**	**Recipient strain**		
	**SC096**	**Δ*fadD1*::Gm^*r*^**	**Δ*fadD2*::Kan^*r*^**
pSF313 (Δ*fadD1*-Gm^*r*^)	Δ*fadD1*::Gm^*r*^	DNC	NA
pSF314 (Δ*fadD2*-Kan^*r*^)	Δ*fadD2*::Kan^*r*^	NA^b^	DNC
pSF324 *(fadD1*^T214A^-Em^*r*^)	DNC^a^	*fadD1*^T214A^-Em^*r*^	NA
pSF324 (*fadD1*^G216A^-Em^*r*^)	DNC	*fadD1*^G216A^-Em^*r*^	*fadD1*^G216A^-Em^*r*^ Δ*fadD2*::Kan^*r*^
pSF324 (*fadD1*^T217A^-Em^*r*^)	DNC	*fadD1*^T217A^-Em^*r*^	NA
pSF324 (*fadD1*^G219A^-Em^*r*^)	DNC	*fadD1*^G219A^-Em^*r*^	*fadD1*^*G*219*A*^-Em Δ*fadD2*::Kan^*r*^
pSF324 (*fadD1*^E362A^-Em^*r*^)	DNC	*fadD1*^E362A^-Em^*r*^	NA

a *DNC, Did not construct*.

b *NA, Not available*.

**Table 5:** **Growth of ***E. coli*** complementation strains on fatty acids of varying chain length**.

**C-source**	**Strains**								
	**JW1794**	**pBAD24m in JW1794**	**pBAD24m-*fadD1* in JW1794**	**pBAD24m-*fadD2* in JW1794**	**pBAD24m-*fadD1*-T214A in JW1794**	**pBAD24m-*fadD1*-G216A in JW1794**	**pBAD24m-*fadD1*-T217A in JW1794**	**pBAD24m-*fadD1*-G219A in JW1794**	**pBAD24m-*fadD1*-E362A in JW1794**
C14:0	−	−	+	+	ND^a^	ND	ND	ND	ND
C16:0	−	−	+	+	ND	ND	ND	ND	ND
C18:1	−	−	+	−	+	+	+	+	−

a *ND, Not determined*.

*E. coli complementation strains were grown on M9 minimal medium plates supplemented with 0.1% oleic acid with Brij58 and results were determined after the cells were incubated for 72 or 120 h at 37°C. Growth was denoted as plus sign if growth after 72 h was observed or minus sign when no colonies were apparent at 120 h of incubation*.

### The effect of *fadD* gene deletion on growth and serum resistance

**Figure 5:**
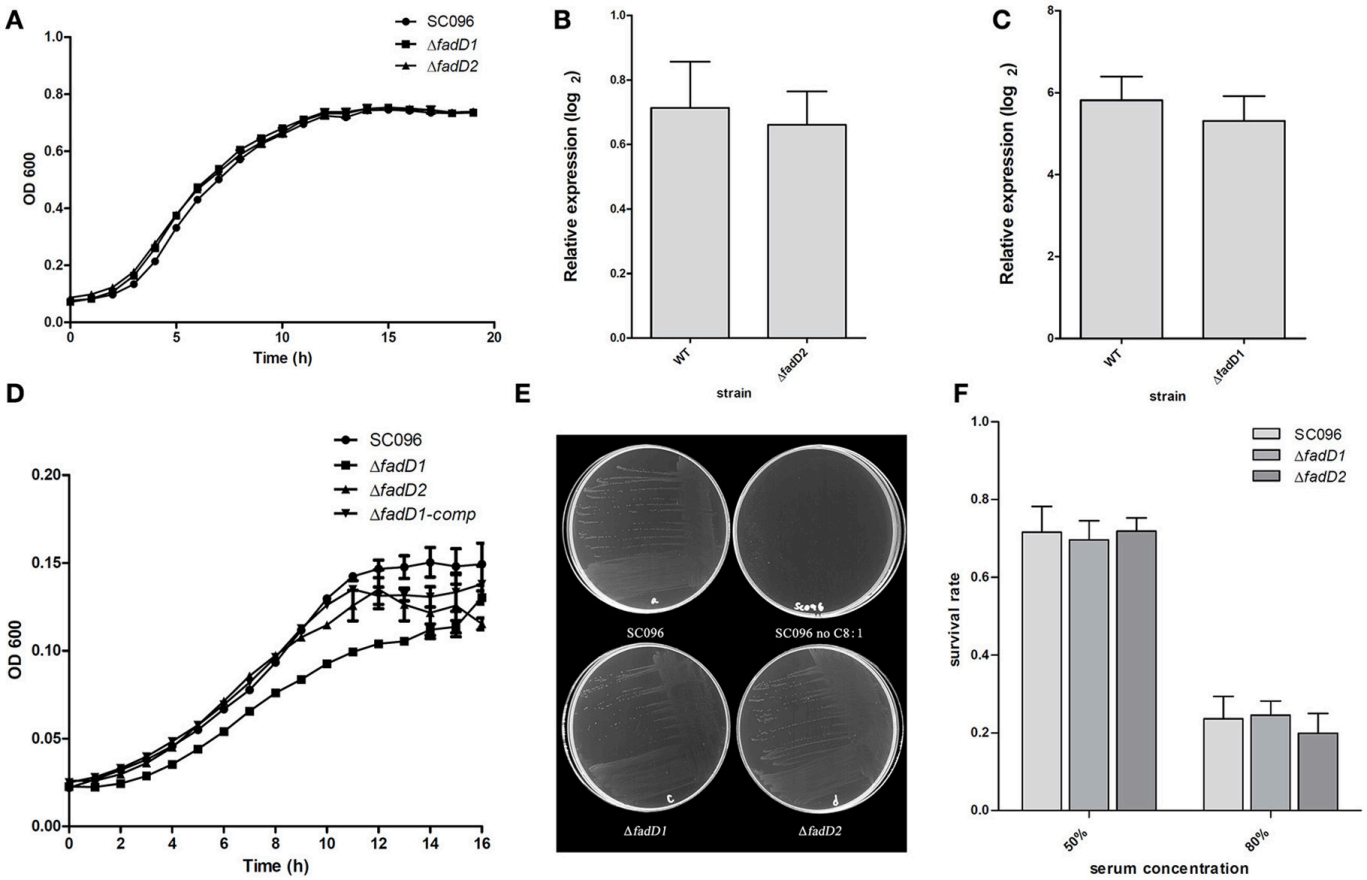
**Analysis of growth, the expression level of ***fadD*** genes and serum resistance of ***H. parasuis fadD*** mutants. (A)** The growth curve of wild type and mutants grown in TSA medium with 5% newborn bovine serum and NAD. Only negligible changes in the growth rates were detected. **(B)** Real-time PCR analysis of *fadD1* transcription in SC096 or Δ*fadD2*. **(C)** Real-time PCR analysis of *fadD2* transcription in SC096 or Δ*fadD1*. Error bars represent the standard deviation from three independent experiments. No significant changes in transcription were observed in real-time PCR assays. **(D)** The growth curve of SC096, *fadD* mutants and *fadD1* complementation strain grown in chemical defined medium with the addition of oleic acid. **(E)** The growth of SC096 and its *fadD* mutants on chemically defined medium plates in either the presence or the absence of oleic acid. **(F)** Survival of SC096 and *fadD* mutant strains in 50 or 80% porcine sera. The data represent means standard errors (*n* = 3) and no statistical difference was detected.

Next, the *fadD1* and *fadD2* mutants were tested to determine their physiological and virulence phenotypes. We tested the growth of the wild-type strain and the mutants in TSB medium containing bovine serum and NAD. Only negligible changes in growth rates were detected in cells grown in this rich medium (Figure 5A). An RT-PCR analysis of total *H. parasuis* RNA extracted from log-phase cells showed that the expression level of *fadD2* was not significantly altered in the *fadD1* mutant (Figure 5B, the relative expression levels of *fadD1* in WT and Δ*fadD2* were 0.71 ± 0.14 and 0.66 ± 0.10, respectively, *P* > 0.05). Similarly, the level of *fadD1* transcription in the Δ*fadD2* strain was equivalent to that in the wild-type strain (Figure 5C, the relative expression levels of *fadD2* in WT and Δ*fadD1* were 5.81 ± 0.58 and 5.31 ± 0.61, respectively, *P* > 0.05). When growth testing was performed using a chemically defined medium (CDM) containing oleic acid, the generation time for the *fadD1* mutant was appreciably longer than that for the wild-type strain SC096 and its complementation strain (Figure 5D, the doubling times of SC096 and the Δ*fadD1* and Δ*fadD1* complementation strains were 3.31 ± 0.17, 4.98 ± 0.07, and 3.93 ± 0.25 h, respectively, *P* < 0.05). Smaller colonies of the *fadD1* mutant grew on CDM agar, and these exhibited a phenotype consistent with the broth medium testing results. Additionally, no visible growth was detected when the wild-type strain was grown on CDM agar without fatty acids, indicating that acyl-CoA may be indispensable for the survival of *H. parasuis* (Figure 5E). Furthermore, the roles of the *fadD* genes in *H. parasuis* serum resistance were investigated. However, there was no significant difference in sensitivity between the wild-type strain and its mutants (Figure 5F, the survival rates of SC096, Δ*fadD1* and Δ*fadD2* in 50% porcine serum were 71.6 ± 6.6, 69.6 ± 4.9, and 71.9 ± 3.4%, respectively. The survival rates of SC096, Δ*fadD1* and Δ*fadD2* in 80% porcine serum were 23.6 ± 5.7, 24.5 ± 3.7, and 19.9 ± 5.1%, respectively, *P* > 0.05).

### FadD1 plays a role in *H. parasuis* quinolone antibiotic resistance

**Figure 6:**
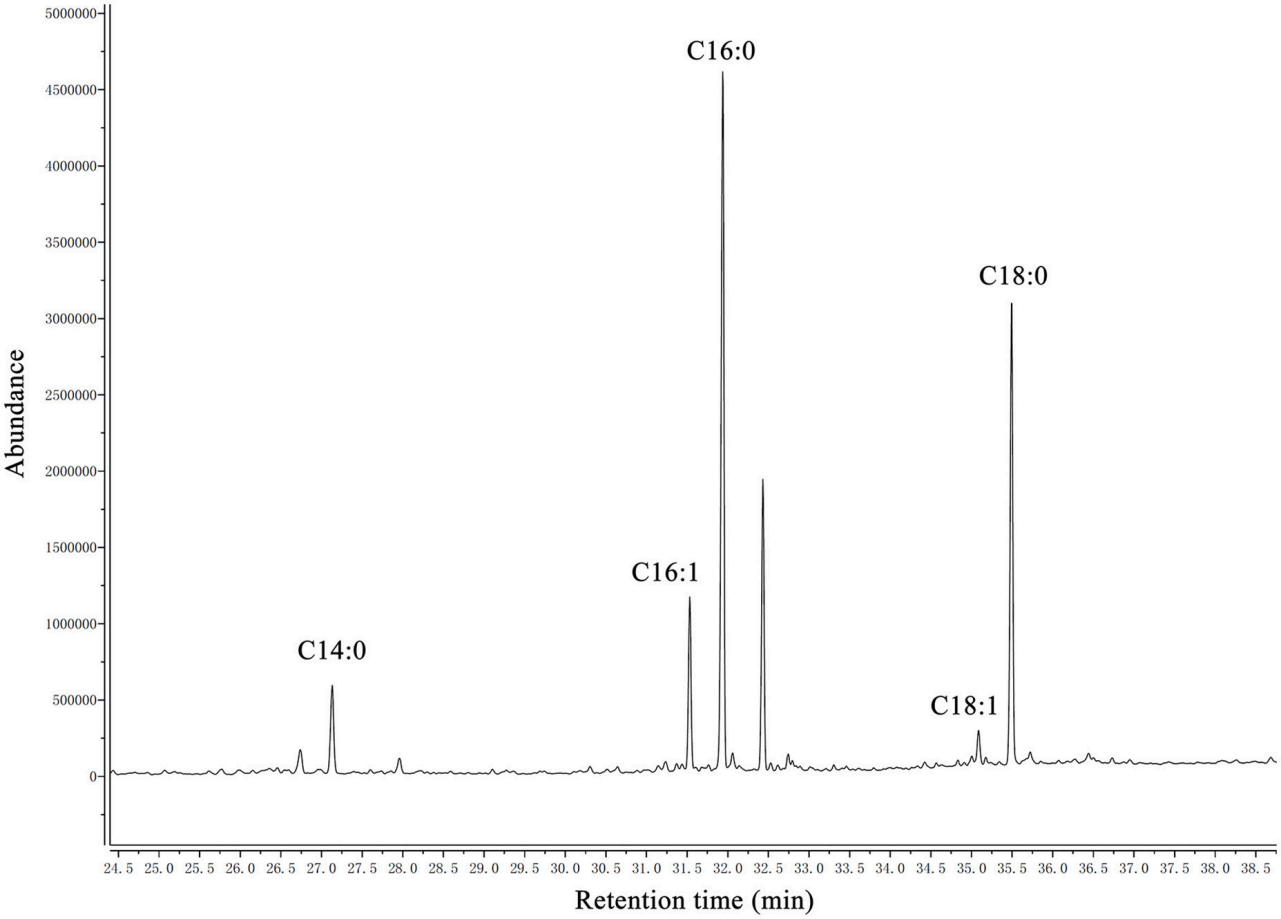
**GC–MS chromatogram of analysis of free fatty acids extracted from phospholipid of ***H. parasuis*** SC096**. The fatty acids were derivatized to their methyl esters and then analyzed by gas chromatography-mass spectroscopy. C14:0, C16:0, C16:1, C18:1, and C18:0 are abbreviations of myristic acid, palmitic acid, palmitoleic acid, oleic acid and stearic acid, respectively.

A summary of the MIC values of several antibiotics that were evaluated in this study is shown in Table 6. Interestingly, the *fadD1* mutant was more susceptible to all of the selected quinolone antibiotics, including levofloxacin, enrofloxacin, nalidixic acid, and ciprofloxacin, whereas the *fadD1* complementation strain exhibited a phenotype similar to the wild-type strain. Note that quinolone resistance can result from changes in bacterial membrane permeation (Neu, 1992). The free acid of nalidixic acid, for instance, is easily dissolved in chloroform but only slightly soluble in water, indicating that both the polarity of quinolone in the medium and the composition of phospholipids (i.e., the proportions of different fatty acids) may influence the diffusion of quinolone through the membrane. To analyze changes in phospholipids, we assayed the fatty acid composition of the cells using GC-MS. The phospholipid fatty acids in *H. parasuis* are composed mainly of palmitic acid, stearic acid, palmitoleic acid, myristic acid and oleic acid (Figure 6). However, no significant differences were observed in fatty acid composition between the wild-type strain and the mutants (Table 7). Hence, we propose that deleting *fadD1* might affect the amount of all lipids rather than the overall composition.

**Table 6:** **Susceptibilities of ***H. parasuis*** strains to antimicrobials**.

**Strain and relevant phenotype(s)**	**MIC (**μ**g/ml)**^a^																	
	**LVX**	**ENR**	**NAL**	**CIP**	**ERY**	**AMP**	**EFT**	**GEN**	**AMK**	**CTX**	**KAN**	**STR**	**SMZ**	**TMP**	**DOX**	**TET**	**FF**	**PB**
SC096	1	1	64	1	1	0.125	≤0.016	2	16	≤0.016	8	16	≥512	4	0.25	0.5	2	0.032
Δ*fadD1*	0.25	0.25	16	0.25	0.25	0.125	≤0.016	32	4	≤0.016	2	8	≥512	4	0.25	0.25	1	0.032
Δ*fadD2*	1	1	64	1	1	0.125	≤0.016	1	16	≤0.016	32	8	≥512	4	0.25	0.25	2	0.032
Δ*fadD1*-comp	1	1	64	2	≥256	0.125	≤0.016	64	16	≤0.016	8	16	≥512	4	0.25	0.25	2	0.032

a *LVX, levofloxacin; ENR, enrofloxacin; NAL, nalidixic acid; CIP, ciprofloxacin; ERY, erythromycin; AMP, ampicillin; EFT, ceftiofur; GEN, gentamicin; AMK, amikacin; CTX, cefotaxime; KAN, kanamycin; STR, streptomycin; SMZ, sulfamethoxazole; TMP, trimethoprim; DOX, doxycycline; TET, tetracycline; FF, florfenicol; PB, polymyxin B*.

**Table 7:** **Fatty acid composition of phospholipid extracts from ***H. parasuis fadD*** mutant strains grown in TSB medium**.

**Fatty acid^a^**	**Strains**				
	**SC096**	**Δ*fadD1***	**Δ*fadD2***	**Δ*fadD1*-comp**	**Δ*fadD1*-comp**
C14:0	6.9 ± 0.5	6.7 ± 0.2	5.3 ± 0.1	4.1 ± 0.7	4.8 ± 0.4
C16:0	48.4 ± 3.2	47.0 ± 4.2	48.8 ± 3.9	48.5 ± 3.7	48.1 ± 2.9
C16:1	11.9 ± 1.8	9.1 ± 0.8	9.3 ± 0.9	10.75 ± 0.6	11.0 ± 1.0
C18:0	29.5 ± 4.2	34.6 ± 2.4	34.7 ± 3.3	34.8 ± 2.5	34.2 ± 2.8
C18:1	3.3 ± 0.3	2.6 ± 0.5	2.0 ± 0.8	1.9 ± 0.4	1.9 ± 0.6

a *The fatty acids listed are those positively assigned by gas chromatographic data and with an abundance of greater than 0.1% in at least one sample. The data presented represent the mean ± S.D. (n = 3) of results from three independent experiments*.

## Discussion

Increases in drug-resistant pathogenic bacteria have created an urgent need for new antibiotics. Type II fatty acid synthesis (FASII) is essential for the formation of cellular membranes in bacteria. There are significant differences between mammalian and bacterial cells that make FASII enzymes efficient targets of antibacterial agents (Campbell and Cronan, 2001). There are currently many examples of natural or artificial products, including cerulenin, cephalochromin and triclosan, that have evolved to target FASII enzymes (Zhang et al., 2006). FASII inhibition cannot be bypassed in many bacteria because they cannot obtain essential fatty acids from the host (Yao and Rock, 2015). However, some pathogens can down-regulate FASII enzymes when exogenous fatty acids are present, allowing them to evade therapeutics. Previous studies have shown that exogenous fatty acids fully bypass inhibition by this pathway under both *in vitro* and *in vivo* conditions in gram-positive pathogens (Brinster et al., 2009). Nevertheless, few studies have focused on acyl-CoA synthetase (FACS) as a target for drug discovery because it may be not essential for the survival of many pathogens. Here, we present evidence showing that *fadDs* (long-chain FACS genes) are essential for *H. parasuis* SC096 survival (Figure 4 and Table 4), suggesting that these genes may be new targets for antibiotics and other drugs aimed at controlling *Haemophilus* bacterial infections. Notably, the human pathogen *Haemophilus influenza* also contains two predicted *fadDs* that share high homology with the *fadDs* in *H. parasuis*, although no studies have explored whether they are essential for *H. influenza*. In addition, the *H. parasuis fadD1* mutant showed increased sensitivity to quinolone antibiotics (Table 6), suggesting that if antibacterial agents could be developed against FadD, drug combinations including these agents may more effectively control these bacterial infections.

In this study, we also focused on characterizing *H. parasuis* FACSs. In *E. coli*, only two FACSs, FadD and FadK, have been identified (Black et al., 1992; Morgan-Kiss and Cronan, 2004), whereas at least six FadD homolog with a wide range of fatty acid activity have been described in *Pseudomonas aeruginosa* (Zarzycki-Siek et al., 2013). The diversity of substrates observed across different FACSs may be due to the evolution of bacteria in different ecological niches. To colonize such diverse environments, bacteria must rapidly adjust their metabolism and defense systems (DiRusso et al., 1999). Unlike many other bacteria, *H. parasuis* has only two FACSs for long-chain activation (Figure 3). The essential nature of *H. parasuis fadDs* may have resulted from adaptations to the host environment that occurred over a long period of evolutionary time. Our results demonstrate that host phospholipids may be vital nutrients to bacteria during *H. parasuis* infection. Additionally, FadD2 activity is sufficient to promote *fadD1* mutant growth without phenotypic differences in rich medium (Figure 5A). The existence of *fadD2* seems to be a supplementary mechanism rather than a strategy for wide-range fatty acid utilization, as the substrate specificity of these two FACSs is similar, although FadD2 exhibited lower activity (Figure 3). Palmitic acid may be a preferred fatty acid substrate according to our data, although a more extensive assessment of potential substrates of FadD2 is required to verify this. It is important to note that the activity of FACS *in vivo* may be determined not only by enzymatic properties but also by expression level. The result that FadD2 can support the growth of a *fadD1* mutant without obvious differences may due to a higher expression level of FadD2 compared with FadD1.

**Figure 7:**
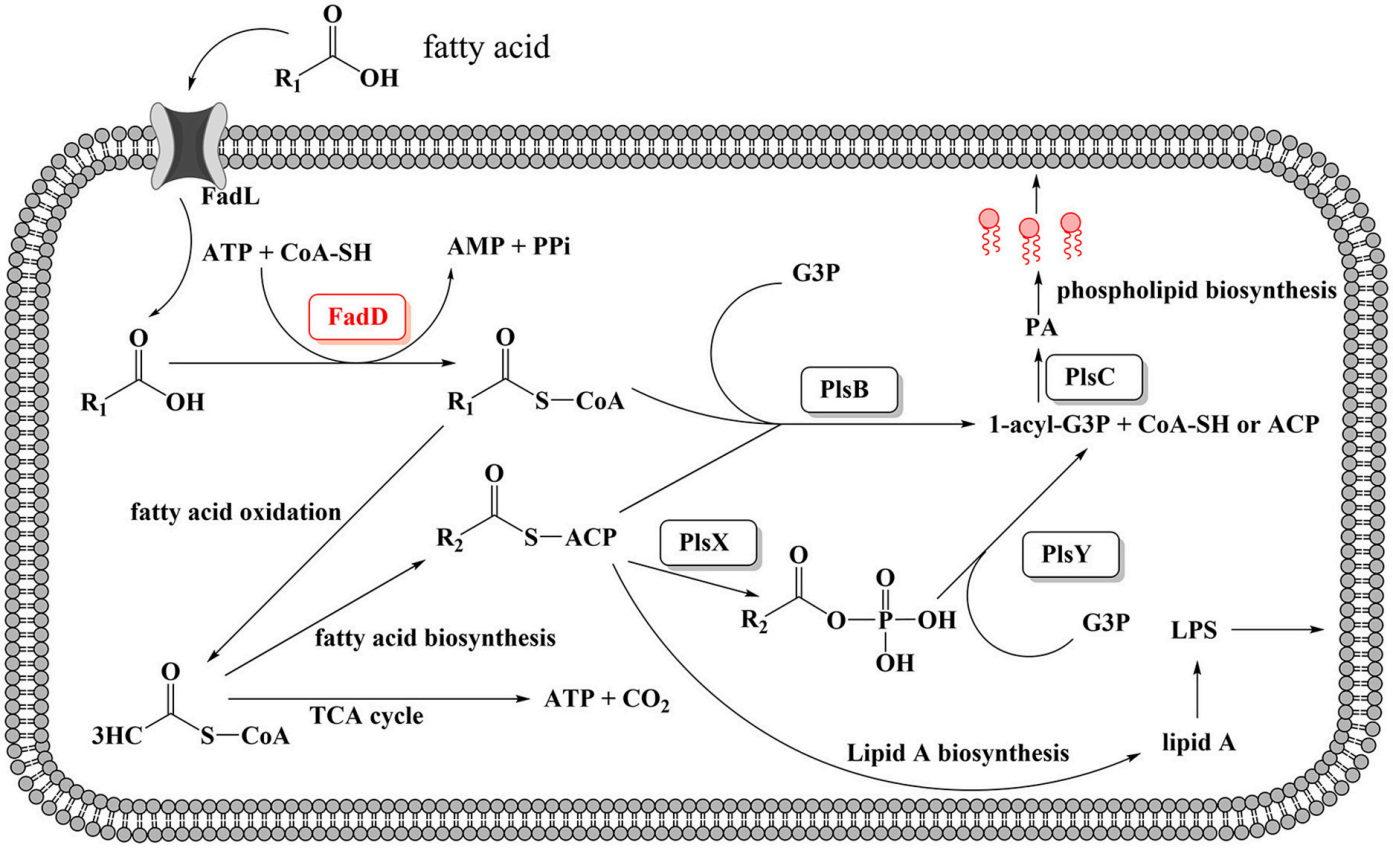
**Proposed model for exogenous fatty acid utilization by ***H. parasuis*** FadDs**. Exogenous fatty acids can be transported through the membrane by an outer membrane protein, for instance, FadL protein. Fatty acids then can be activated by acyl-CoA synthetase and converted to acyl-CoA. For *H. parasuis*, the enzymes may be FadD1 and FadD2. Subsequently, acyl-CoA can either be degraded to acetyl-CoA through β-oxidation or be utilized by PlsB and PlsC to synthesize the phospholipids then incorporate to the membrane. It should be noted that endogenous fatty acid is essential and can be synthesized from acetyl-CoA by FAS, then used for lipid A or phospholipids biosynthesis.

A schematic diagram illustrates the exogenous fatty acid metabolism (Figure 7). It must be noted that while exogenous fatty acids can be incorporated into membrane phospholipids, they can also be converted to acetyl-CoA, which is a precursor for *de novo* fatty acid biosynthesis. There is no pathway through which exogenous fatty acids can be converted into β-hydroxyacyl-ACP, which is required for lipid A biosynthesis; lipid A is the core component of LPS, which is required for bacterial survival (Yao and Rock, 2015). Previous studies have shown that lipooligosaccharide (LOS) is an *H. parasuis* virulence factor. Mutants with truncated LOSs exhibit decreased resistance to complement-mediated killing in serum (Xu et al., 2013; Zhou et al., 2016). The results presented in this report demonstrate that the wild-type strain SC096 cannot grow on chemically defined medium without the addition of fatty acids (Figure 5E). Fatty acids and acyl-CoA are therefore indispensable, even though other carbon sources, such as amino acids and vitamins, are present in this medium. Exogenous fatty acids may be mainly converted to acetyl-CoA rather than directly transferred to the membrane. This process is followed by the rebuilding of phospholipids and lipid A via the FAS pathway. Hence, the fatty acid composition of phospholipids or lipid A may be determined by FAS but not the types or amount of exogenous fatty acids. This may explain why deleting *fadD1* had no significant effect on fatty acid composition or serum resistance (Figure 5F and Table 7). Further studies are needed to confirm how disruptions in FadDs affect the *H. parasuis* membrane and LOS.

## Conclusions

In summary, we identified two FACS homolog and determined the substrate specificities of each FadD in *H. parasuis*. Mutant and site-directed mutagenesis-generated strains were used to show that either *fadD1* or *fadD2* is required for survival in *H. parasuis*. Moreover, growth on chemical defined medium was affected in this bacterium by the deletion of *fadD1*. Finally, the *fadD1* mutant showed increased sensitivity to quinolone antibiotics.

## Author contributions

SF: Performed research, analyzed data, and wrote the paper. CX: Performed research. KY: Helped with experiment. HW: Analyzed data. HF: Funded research and analyzed data. ML: Funded research and analyzed data.

### Conflict of interest statement

The authors declare that the research was conducted in the absence of any commercial or financial relationships that could be construed as a potential conflict of interest.
