# Supplementary material for: Either fadD1 or fadD2, Which Encode acyl-CoA Synthetase, Is Essential for the Survival of Haemophilus parasuis SC096
Source: Front Cell Infect Microbiol. 2017 Mar 15;7:72. doi: 10.3389/fcimb.2017.00072 (PMC5350145; doi:10.3389/fcimb.2017.00072)
Supplement: Supplementary file 1 [file Image1.PDF]

A

1 MEK**IWFDNYP AEAER**TLDVE PYESLVEMFE KAVQR**HPDIP AYINMGQVLT**

51 **FR**KLEERSR**A FAAYLQNELR** LEKGERIALM IPNLLQYPIA LFGALR**AGLV**

101 **VVNVNPLYTP R**ELEYQLNDS GAKAIVVVSN FAATLEKVVF NTQVKHVILT

151 RMGDQLSFGK RTLVNFVVKY VKKLVPKYKL PHAVSFRETL SIGKQR**QYVR**

201 **PTLYK**NDLAF LQYTGGTTGV AKGAMLSHSN VIANILQAK**W VAYPLIQR**SQ

251 ERIGVIALPL YHVFALTVNC LLFIELGVTG LLITNPRDIP AFVKELKKYP

301 VMAITGVNTL FNALLNNPQL KEVDFSNLKL SVGGGAAIQR AVADRWHK**TT**

351 **GCHIIEGYGM TECSPLIAAT RNDSTEYSGS IGVPVPNTDI RIVDDAGNDL**

401 **PIGER**GELWV K**GPQVMQGYW QRPEDTAEVL KDGWMATGDI VELGQDLNLR**

451 IVDRKKDMII VSGFNVYPNE IEDVVALHPK VNEVVVVGIP SEVSGESIKV

501 FVTKKDESLT REELRNHCRQ HLTGYKIPRE IEFRDELPKS NVGKILRRVL

551 RDEEVARVKS QMEGKL

B

1 M**ASLDFHFVN R**FRLQAKKWL NRTALRFR**EQ AQWQEMSWQT FQQEIDR**FSY

51 ALIAQHIDIQ DKIGIFANNM PRWTIADFGA MQARAVAVPI YATNTAKQVE

101 YIVNDADIKI LFVGDQEQLD QVCQIANNCP QLMKIVAMKA NMDLRDLPNA

151 CYWEDFLDVV PNEAEFEKRL NSKQLSDLFT LIYTSGTTGE PKGVMLDYAN

201 LAHQLNAHDL ALNVNEDDVS LSFLPLSHIF ER**AWVAYVFH RGATNCYLED**

251 **TNHVR**DALTT LKPTVMCAVP RFYEKIYTAV WDKVEKAPAH RR**ALFNWAIR**

301 VGEK**HYQTEQ PSQWLR**LQYA LADKLVLTKL RALLGGRIKM MPCGGAKLEA

351 SIGSFFHSIG INIKLGYGMT ETTATVSCWQ DKGFNPNSIG TLMPNAEVK**I**

401 **GEENEILVR**G GMVMRGYYKK PEETAK**AFTE DGFLR**TGDVG EMDSCGNLFI

451 TDRLKELMKT LNGKYIAPQY IEGKIGKDKF IEQIAVIADA KKYVSALIVP

501 CFDSLEEYAK QLNIKYQDRI ELIKHSDIIQ MFERRIHELQ KELPSFEQVK

551 KFTLLPQAFS TAMEEITPTL KLRRKVIMQR YREQIEEMYN ERSLT

Fig. S1. Mass spectrometry identification of FadDs. The matching peptides are given in bold and marked as red. (A) FadD1 and (B) FadD2

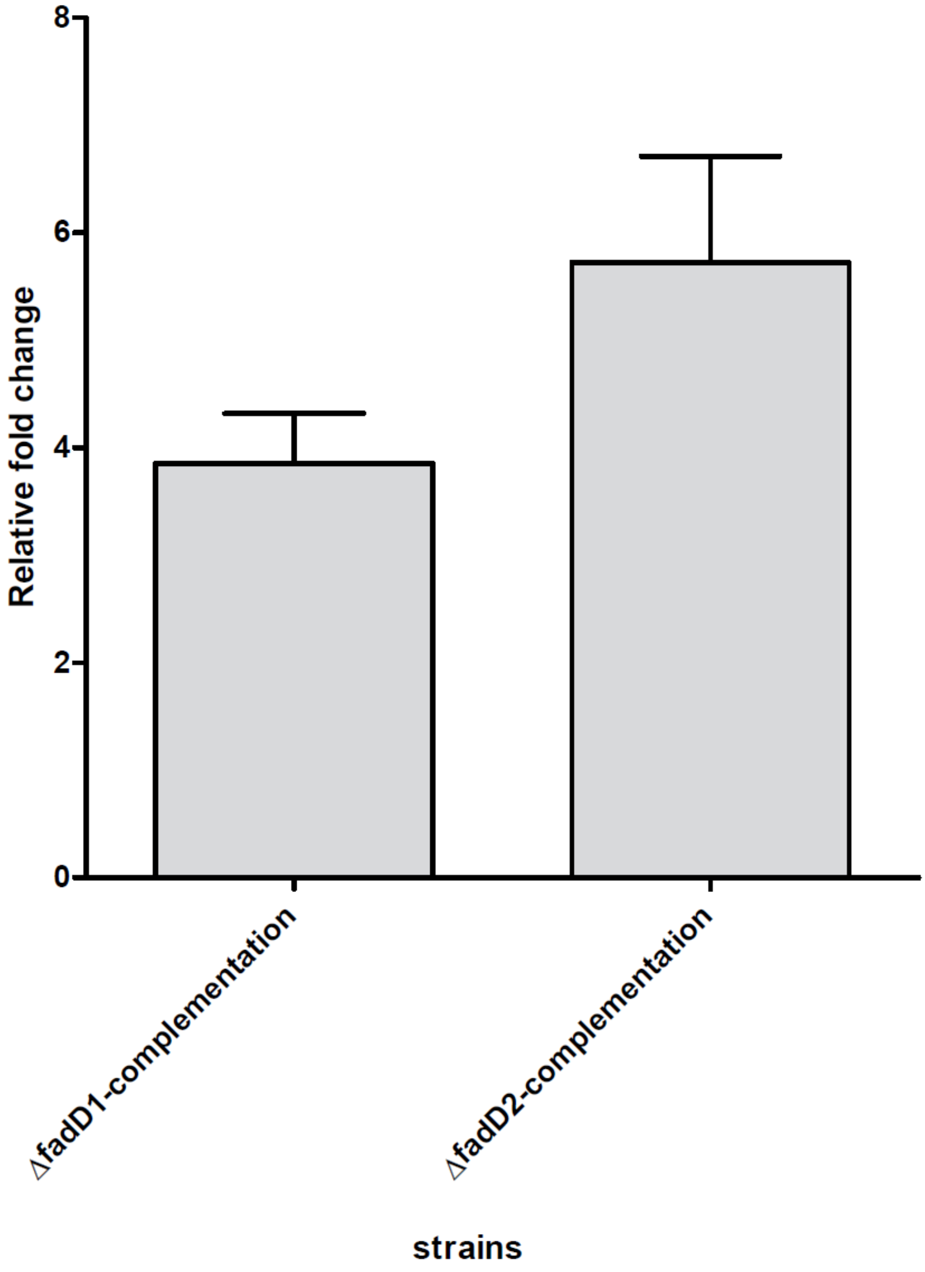

Fig. S2. Expression of *fadD1* in Δ*fadD1* complementation strain and *fadD2* in Δ*fadD2* complementation strain. Normalized with expression of *fadD1* or *fadD2* in wild-type strain respectively. Error bars represent the standard deviation from three independent experiments.
